# Effects of omega‐3 supplementation on psychological symptoms in men with prostate cancer: Secondary analysis of a double‐blind placebo‐controlled randomized trial

**DOI:** 10.1002/cam4.6598

**Published:** 2023-10-03

**Authors:** Josée Savard, Hanane Moussa, Jean‐François Pelletier, Pierre Julien, Louis Lacombe, Rabi Tiguert, Yves Caumartin, Thierry Dujardin, Paul Toren, Frédéric Pouliot, Michele Lodde, Yves Fradet, Karine Robitaille, Vincent Fradet

**Affiliations:** ^1^ School of Psychology Université Laval Québec Canada; ^2^ CHU de Québec‐Université Laval Research Center Québec Canada; ^3^ Université Laval Cancer Research Center Québec Canada; ^4^ Faculty of Medicine Université Laval Québec Canada; ^5^ Institute of Nutrition and Functional Foods (INAF) and Nutrition Health and Society (NUTRISS) center of Université Laval Québec Canada

**Keywords:** anxiety, cognitive impairments, depression, fatigue, fear of cancer recurrence, insomnia, omega‐3, prostate cancer, randomized clinical trial

## Abstract

**Background:**

In the general population, a higher omega‐3 polyunsaturated fatty acids intake is associated with lower levels of several psychological symptoms, especially depression. However, the existing evidence in cancer is equivocal.

**Methods:**

This phase IIB double‐blind, placebo‐controlled trial was aimed at comparing the effects of eicosapentaenoic acid monoacylglyceride (MAG‐EPA) supplementation and high oleic acid sunflower oil (HOSO; placebo) on depression levels (primary outcome) and other symptoms (anxiety, fear of cancer recurrence, fatigue, insomnia, perceived cognitive impairments; secondary outcomes). Participants, recruited in a prostate cancer clinic, were randomized to MAG‐EPA (3.75 g daily; *n* = 65) or HOSO (3.75 g daily; *n* = 65) for 1 year post‐radical prostatectomy (RP), starting 4–10 weeks before surgery. Patients completed self‐report scales at baseline (before RP) and 3, 6, 9, and 12 months after: Hospital Anxiety and Depression Scale (HADS), Fear of Cancer Recurrence Inventory (FCRI), Insomnia Severity Index (ISI), Fatigue Symptom Inventory (FSI), and Functional Assessment of Cancer Therapy—Cognitive Function (FACT‐Cog).

**Results:**

Analyses showed significant reductions in HADS‐depression, HADS‐anxiety, FCRI, ISI, FSI‐number of days, and FACT‐Cog‐impact scores over time. A significant group‐by‐time interaction was obtained on FACT‐Cog‐Impact scores only; yet, the temporal change was significant in HOSO patients only.

**Conclusions:**

Several symptoms significantly decreased over time, mainly within the first months of the study. However, MAG‐EPA did not produce greater reductions than HOSO. Omega‐3 supplementation does not seem to improve psychological symptoms of men treated with RP.

## 1 INTRODUCTION

Prostate cancer is the most widely diagnosed non‐skin malignancy in North American men.^1^, ^2^ Although prostate tumors generally have a good prognosis,^3^ their treatment with radical prostatectomy (RP) or radiation therapy is associated with significant morbidity (e.g., erectile dysfunction, incontinence), which may take a significant toll on patients' psychological health. A meta‐analysis of 27 studies (*N* = 4494) showed that 17.3%, 14.7%, and 18.4% of patients with prostate cancer had clinical levels of depression at pretreatment, during treatment, and posttreatment, respectively, as assessed using validated questionnaires.^4^ Proportions of patients with clinical anxiety were 27.0%, 15.1%, and 18.5% at the same time points, respectively. Fatigue, insomnia, and cognitive disturbances are even more common in men with prostate cancer. The prevalence of chronic fatigue varies from 13% (RP) to 39% (radiation + hormone therapy).^5^ The prevalence of insomnia symptoms ranges from 25% to 50%, with higher values found at the perioperative period.^6^ Cognitive impairments are found in 10%–69% of patients, depending on measures used, domains evaluated, treatments received and time since their termination.^7^

In the general population, epidemiological studies have consistently found associations between a greater fish consumption and omega‐3 (n‐3) polyunsaturated fatty acids (PUFA) intake and a reduced risk for depression,^8^, ^9^ with a peak reduced risk for 1.8 g/day of n‐3 and 0.6 g/day of EPA + DHA intake.^8^ There is also some evidence of a linear relationship between plasma levels of n‐3 and the severity of depression in depressed patients.^10^, ^11^, ^12^ Further, reviews of randomized clinical trials (RCTs) have shown a greater reduction of depressive symptoms with n‐3 PUFA supplementation as compared to placebo,^13^, ^14^ with greater effects for eicosapentaenoic acid (EPA) than docosahexaenoic acid (DHA)‐predominant formulations (>50%), at a dosage of 1 g/day or more.^14^, ^15^ For sleep difficulties, RCTs have mainly revealed no beneficial effect in menopausal women (12 weeks of 1.8 g/day fish oil capsules, 3 times/day; containing 425 mg of EPA, 100 mg of DHA and, 100 mg of other n‐3 s) and middle‐aged and older adults (12 weeks of 2.5 g/day, 2085 mg of EPA and 348 mg of DHA capsules).^16^, ^17^ However, reviews of RCTs have revealed promising results for the treatment of anxiety disorders (for dosages of at least 2000 mg/day and for supplements containing at least 60% or more EPA)^18^ and for improving cognitive functioning in the elderly.^19^

A handful of studies have looked at relationships between n‐3 PUFA consumption/levels and psychological symptoms in patients with cancer. In lung cancer, no significant difference was found on serum levels of n‐3 PUFA between patients with severe major depression and nondepressed patients, but the group with minor depression had significantly higher DHA levels than the other two.^20^ On the other hand, a study of patients with breast cancer showed a relationship between greater depression scores and higher blood levels of total n‐6 PUFA and α‐linolenic acid (ALA), but not n‐3 levels.^21^ Using the same sample, lower blood levels of ALA, but not EPA and DHA, were significantly related to higher fear of cancer recurrence (FCR), independently of depressive symptoms.^22^ With regard to cognitive function, it has been suggested that a diet rich in n‐3 PUFA, especially DHA, could help prevent impairments associated with cancer treatment (e.g., chemotherapy), but evidence is lacking.^23^, ^24^ Available RCTs of n‐3 supplementation (6‐week treatment of 1‐g capsules containing 18% EPA and 12% DHA^25^ or oral nutritional supplement containing EPA^26^) have shown reductions in fatigue in patients with advanced lung cancer. A pilot RCT also found that a 3‐month diet rich in fruits, vegetables, whole grains, and n‐3 PUFA was associated with reduced fatigue and improved sleep in patients with breast cancer.^27^ However, a recent 6‐week trial showed that fatigue improvements were greater with n‐6 (capsules of soybean oil; 6 g/day) than n‐3 (capsules of fish oil; 3.3 g/day of DHA + EPA) supplementation in the context of breast cancer.^28^

In sum, the existing evidence, mainly cross‐sectional, has provided equivocal findings on the link between PUFA intake/levels and several symptoms in the context of cancer. More RCTs are needed to establish causality, and no study has been conducted in the context of prostate cancer. This study is a secondary analysis of an RCT assessing the effects of eicosapentaenoic acid monoacylglyceride (MAG‐EPA) supplementation on biological and quality of life outcomes in men treated with RP for prostate cancer (Gleason score ≥7 and grade group ≥2^29^, ^30^). This analysis was planned a priori at the time of the grant proposal submission, hence before the start of the trial. The main outcome of this RCT was the proliferative index (nuclear Ki‐67 expression) of prostate cancer cells from RP specimen. The goal of the current secondary outcome analysis was to evaluate the effect of a 1‐year MAG‐EPA (active treatment) supplementation as compared to high oleic acid sunflower oil (HOSO; placebo) on depression (main outcome) and other psychological symptoms* commonly found in men with prostate cancer (anxiety, FCR, insomnia, fatigue, and cognitive complaints). It was hypothesized that patients taking MAG‐EPA supplementation would show significantly greater reductions in depression and other symptoms as compared to HOSO at 3‐, 6‐, 9‐, and 12‐month follow‐ups.

## 2 METHOD

### 2.1 Study design

The study is a phase IIb, randomized, double‐blind, placebo‐controlled trial. A total of 130 patients were randomized (allocation ratio 1:1) to MAG‐EPA supplementation (*n* = 65) or HOSO (*n* = 65) 4–10 weeks before undergoing their RP. Psychological measures were collected at baseline (V0), as well as 3 (V2), 6 (V3), 9 (V4), and 12 (V5) months following RP. No psychological measure was taken at RP (V1). This sample size was estimated to provide sufficient power to detect significant between‐group differences on the main RCT outcome (i.e., proliferative index).

### 2.2 Participants

#### 2.2.1 Inclusion criteria

(a) ≥18 years of age; and (b) waiting to receive RP for prostate cancer with a Gleason score ≥7 (grade group ≥2).

#### 2.2.2 Exclusion criteria

(a) Intolerance or allergy to fish or sunflower seeds; and (b) antecedents of bipolar disorder (as reported by the patient and corroborated in his medical chart). Users of n‐3 supplements could be included after a washout period of 8 weeks prior to randomization (three patients did so). Other types of natural supplements had to be stopped throughout the study.

#### 2.2.3 Recruitment

**FIGURE 1:**
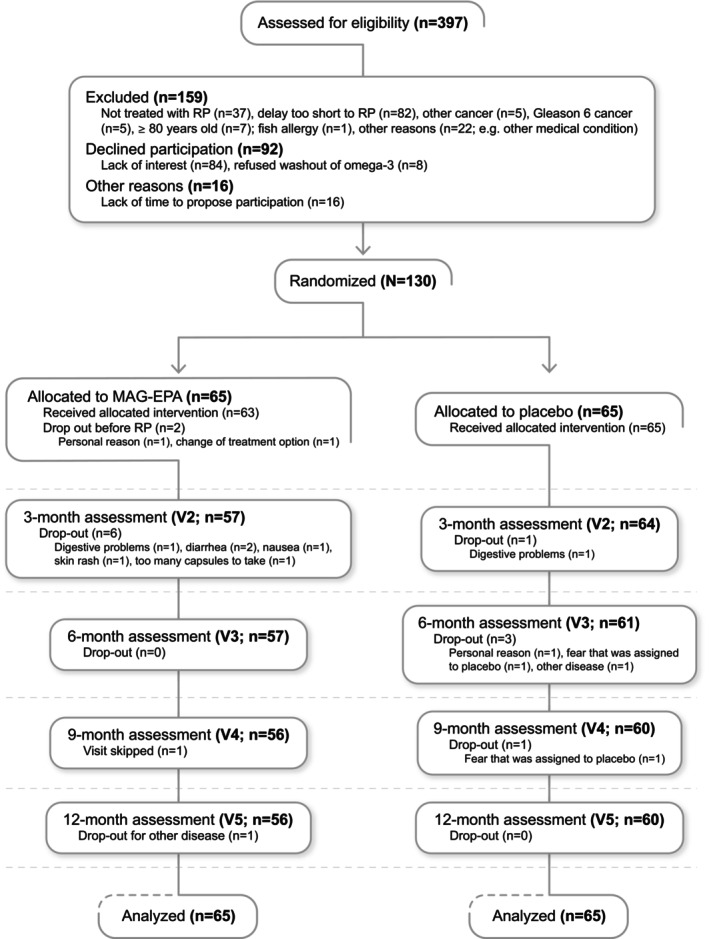
Participants' flowchart.

The trial was advertised through posters in the prostate cancer clinic of the CHU de Québec‐Université Laval. When the prostate cancer diagnosis was ascertained, the urologist informed the patient about the various treatment options. When RP was selected as the primary treatment, the urologist briefly informed the patient about the study and referred him to a research nurse who explained it in more detail and obtained written consent of patients agreeing to participate. Between February 2015 and June 2017, 397 patients were assessed for eligibility, and 130 of them (32.7%) agreed to participate and were randomized (see Figure 1). The trial was approved by our Institution's Review Board (#2012‐1012).

#### 2.2.4 Randomization

Participants were randomized after their eligibility was confirmed, at their enrolment visit, which occurred 4–10 weeks prior to their RP. A computer‐generated random list was prepared by the clinical research oncology pharmacy (permuted random blocks of 2–8) and was kept locked at the pharmacy. All patients and members of the research team were blinded to the allocation sequence and block sizes.

### 2.3 Procedure

#### 2.3.1 Assessments

At randomization, blood samples and anthropometry measures were taken. Patients also completed self‐report scales assessing medical history, health behaviors, psychological and somatic symptoms, and quality of life. Patients had to come back to the clinic for a nurse appointment 3, 6, 9, and 12 months following RP when they completed the same battery of questionnaires and samples collection.

#### 2.3.2 Intervention

Both types of capsule were prepared by SCF Pharma, were odorless, and were identical in appearance. Patients were instructed to take the capsules 7.5 weeks on average (range: 4–10 weeks) prior to RP and to continue for 1‐year post‐RP. At each visit, they were given enough capsules for 3 months by the hospital pharmacist and were instructed to return those not taken since the last visit. No major toxicities were observed for both arms. Adverse events have recently been published in another secondary analysis (quality of life outcomes) of the same trial.^31^

*MAG‐EPA* (*active intervention*)

These patients were instructed to take six capsules of 625 mg of fish oil (each containing 500 mg of MAG‐EPA) per day for a total daily dosage of 3 g of EPA. This intervention was chosen based on our previous studies in men with low‐risk prostate cancer which showed that a higher EPA level in prostate tissue, but not DHA, was related to a lower cancer progression risk.^32^, ^33^ The dosage of 3 g was also consistent with Health Canada's recommendation to maintain good health at the time of the study.^34^

*HOSO* (*placebo*)

This group had to take six capsules of 625 mg of high oleic acid sunflower oil (mainly n‐9, with no n‐3, and only a trace of n‐6), a biologically neutral oil on inflammation, for a total daily dose of 3.75 g of HOSO.

#### 2.3.3 Measures

*Hospital Anxiety and Depression Scale* (*HADS*)

This 14‐item scale is divided into two subscales: depression (HADS‐D; 7 items) and anxiety (HADS‐A; 7 items).^35^ Items are rated in reference to the previous week on a 4‐point Likert scale ranging from 0 to 3 (total score range: 0–21). A total score ≥7 on a subscale indicates a clinical level of that symptom.^36^

*Patient Health Questionnaire‐9* (*PHQ‐9*)^37^

This self‐report scale comprises nine items assessing depression symptoms. Items are rated on a 4‐point Likert scale from 0 “not at all” to 3 “nearly every day” over the past 2 weeks (total score range: 0–27). A score ≥10 suggests the presence of moderate depression severity.

*Fear of Cancer Recurrence Inventory‐Severity Subscale* (*FCRI‐S*)

The FCRI‐S evaluates the severity of FCR over the past month. It is composed of nine items rated on a 5‐point Likert scale with a range from 0 “not at all” to 4 “a great deal”.^38^ A score ≥13 indicates a clinical level of FCR.^39^

*Insomnia Severity Index* (*ISI*)

The ISI evaluates insomnia severity.^40^ Each of the seven items is rated using a 5‐point Likert scale that varies from 0 “not at all” to 4 “very much”; total range score: 0–28. A score ≥8 suggests the presence of clinically meaningful insomnia.^41^

*Fatigue Symptoms Inventory* (*FSI*)

The FSI was developed in patients with cancer to assess multidimensional aspects of fatigue.^42^ It evaluates fatigue severity (3 items), fatigue impact (7 items), the number of days that respondents felt fatigued (1 item), and how much of the day (extent; 1 item) they felt fatigued. A mean score of 3 or higher on the severity subscale indicates the presence of a clinical level of fatigue.^43^

*Functional Assessment of Cancer Therapy—Cognitive Function* (*FACT‐Cog v3*)

The FACT‐Cog is subdivided into four subscales: perceived cognitive impairments (PCI; 18 items), comments from others (others; 4 items), impact on quality of life (impact; 4 items), and perceived cognitive abilities (PCA; 7 items). Items are scored in reference to the past 7 days on a 5‐point Likert scale that ranges from 0 “not at all” to 4 “very much”. A PCI score <54 indicates the presence of clinically meaningful cognitive impairments.^44^

### 2.4 Statistical analyses

Groups were compared at baseline on their sociodemographic and medical characteristics using *t*, Cochran–Mantel–Haenszel and Wilcoxon tests (see Table 1). To compare the effects of MAG‐EPA supplementation to those of placebo, group, time, and group‐by‐time interactions were tested using linear mixed models with repeated measures, including a random effect for patients, on each outcome. Because of significant between‐group differences at baseline on these variables, the analyses controlled, when appropriate (i.e., when the confounding variable was significantly associated with the outcome), for the possible confounding effect of the National Comprehensive Cancer Network (NCCN) risk classification (ISI), the Gleason score at biopsy (FCRI, FSI‐severity, FSI‐number of days, and FSI‐extent), and baseline EPA levels (HADS‐A, FSI‐impact, and FACT‐Cog‐PCA). All analyses were conducted using an intent‐to‐treat approach. To test group, time and group‐by‐time interaction effects on the proportion of patients having clinical levels of each symptom, generalized linear mixed models (binomial distribution, logit link) with repeated measures, including a random effect for patients, were computed. The analyses controlled for the same possible confounding variables, when appropriate (biopsy Gleason score for FSI). To investigate whether the evolution of mean scores obtained on a specific symptom for both groups varied as a function of having a clinical level of that same symptom at baseline, linear mixed models with repeated measures were computed, including a random effect for patients, and controlling for the same possible confounders (EPA for FCRI, biopsy Gleason score for ISI, NCCN risk for FACT‐Cog). All double and triple interaction effects between the clinical level of the symptom, the group, and time were included in these models. For the first two series of analyses, *per‐protocol* analyses were also conducted including only the patients who took at least 80% of their capsules between one time point and the previous one (V0: 130; V2: 101; V3: 106; V4: 108; V5: 106). As the pattern of results was quite similar, we only report here findings obtained with all available data.

**TABLE 1:** Participants' characteristics at baseline.

Variable	MAG‐EPA (*n* = 65)	Placebo (*n* = 65)	Total sample (*n* = 130)	*p*
Age (mean, SD)	64.28 (6.22)	62.51 (7.37)	63.39 (6.85)	0.14^b^
Education, *n* (%)				0.73^c^
High school or less	24 (36.92)	19 (29.23)	43 (33.08)	0.42^e^
Postsecondary diploma	18 (27.69)	25 (38.46)	43 (33.08)	
University degree	21 (32.31)	20 (30.77)	41 (31.53)	
Missing	2 (3.08)	1 (1.54)	3 (2.31)	
PSA at randomization (ng/mL; mean, SD)	8.74 (9.33)	6.64 (5.53)	7.69 (7.71)	0.19^d^
Biopsy Gleason, *n* (%)				0.03^e^
7 (3 + 4), grade group 2	31 (47.69)	41 (63.08)	72 (55.38)	
7 (4 + 3), grade group 3	17 (26.15)	18 (27.69)	35 (26.92)	
8, grade group 4	13 (20.00)	5 (7.69)	18 (13.85)	
9, grade group 5	4 (6.15)	1 (1.54)	5 (3.85)	
Cancer stage *n* (%)				0.07^e^
T2a or less	52 (80.00)	59 (90.77)	111 (85.38)	
T2b or T2c	4 (6.15)	3 (4.62)	7 (5.38)	
T3 or more	9 (13.85)	3 (4.62)	12 (9.23)	
NCCN risk, *n* (%)				0.04^e^
Intermediate	45 (69.23)	55 (84.62)	100 (76.92)	
High	20 (30.77)	10 (15.38)	30 (23.08)	
Total n‐3^a^ (mean, SD)	7.40 (1.17)	7.40 (1.02)	7.40 (1.09)	0.98^b^
LCn‐3^a^ (mean, SD)	7.10 (1.16)	7.11 (1.02)	7.10 (1.09)	0.94^b^
EPA^a^ (mean, SD)	0.73 (0.23)	0.80 (0.25)	0.76 (0.24)	0.03^d^
Total n‐6^a^ (mean, SD)	26.44 (1.48)	26.61 (1.39)	26.52 (1.43)	0.49^b^
n‐3/n‐6 ratio^a^ (mean, SD)	0.28 (0.06)	0.28 (0.05)	0.28 (0.06)	0.82^b^

Abbreviations: LCn‐3, long chain omega‐3 polyunsaturated fatty acids; MAG‐EPA, monoacylglyceride‐conjugated eicosapentaenoic acid; n‐3, omega‐3 polyunsaturated fatty acids; n‐6, omega‐6 polyunsaturated fatty acids; NCCN, National Comprehensive Cancer Network; PSA, prostate‐specific antigen.

^a^ Fatty acid profile in red blood cell membranes of fasted patients is expressed as a percentage of total fatty acids, and measured at study baseline using gaz‐chromatography mass spectrometry.

^b^ T‐test.

^c^ Cochran‐Mantel–Haenszel test excluding missing values.

^d^ Wilcoxon test.

^e^ Cochran‐Mantel–Haenszel test.

## 3 RESULTS

### 3.1 Participants' characteristics

On average, participants were 63.4 years old (range: 41–78; see Table 1). A majority was treated for a Stage T2a or less tumor (85.4%), had a biopsy Gleason score of 7 (grade group 2; 55.4%), and an intermediate NCCN risk classification (76.9%). Patients assigned to placebo had significantly greater biopsy Gleason scores (*p* = 0.03), tumor aggressiveness (NCCN risk; *p* = 0.04), and greater EPA levels (*p* = 0.03) at baseline than the MAG‐EPA group. No other significant between‐group differences were found. The proportions of patients with clinical levels of each symptom at baseline are shown in Table 2.

**TABLE 2:** Proportion of patients with clinical levels of symptoms at baseline.

Variable	MAG‐EPA (*n* = 65; %)	Placebo (*n* = 65; %)
HADS‐D (≥7)	9.5	13.9
PHQ‐9 (≥10)	6.3	1.5
HADS‐A (≥7)	25.0	36.5
FCRI‐Severity (≥13)	32.3	53.1
ISI (≥8)	34.4	43.8
FSI‐Severity (≥3)	44.4	48.4
FACT‐COG‐PCI (<54)	22.2	12.9

Abbreviations: FACT‐Cog, Functional Assessment of Cancer Therapy—Cognitive Function; FCRI, Fear of Cancer Recurrence Inventory; FSI, Fatigue Symptom Inventory; HADS‐A, anxiety subscale of the Hospital Anxiety and Depression Scale; HADS‐D, depression subscale of the Hospital Anxiety and Depression Scale; ISI, Insomnia Severity Index; MAG‐EPA, monoacylglyceride‐conjugated eicosapentaenoic acid; PCI, perceived cognitive impairment; PHQ‐9, Patient Health Questionnaire‐9.

### 3.2 Treatment effects on psychological variables (mean scores)

Linear mixed models with repeated measures revealed significant time effects on HADS‐D, HADS‐A, FCRI, ISI, FSI‐number of days, and FACT‐Cog‐impact scores (see Table 3). Post hoc analyses revealed a significant reduction in HADS‐A scores between V0 (*M* = 5.66) and all subsequent time points (*M* = 4.07, 3.7, 3.65, 3.78, respectively), a significant decline of FCRI scores between V0 and V2 (*M* = 11.68 and 10.47, respectively) and subsequent time points (*M* = 8.85, 8.27, 8.48, respectively), and a significant decrease of ISI scores from V0 (*M* = 7.00) and V2 (*M* = 6.87) to V4 (*M* = 5.59). However, no significant differences across time points were found on HADS‐D and FSI‐number of days scores after using a Bonferroni correction for multiple comparisons. No significant group, time, or interaction effect was obtained on PHQ‐9 scores (*p*s = 0.74, 0.10, 0.48, respectively) and other subscales of the FSI and the FACT‐Cog.

**TABLE 3:** Least Squares Means (standard errors) of Scores Obtained on Each Symptom at Each Time Assessment and Results of Mixed Models Analyses of Variance (ANOVAs).

			ANOVAs		
	MAG‐EPA (*n* = 65)	Control (*n* = 65)	Group	Time	Group X time
Variable	*M* (SE)	*M* (SE)	*F*	*F*	*F*
HADS‐D (0–21)			0.03	3.13*	0.84
V0	2.8 (0.39)	3.2 (0.37)			
V2	3.0 (0.33)	2.6 (0.34)			
V3	2.5 (0.30)	2.2 (0.34)			
V4	2.2 (0.33)	2.4 (0.31)			
V5	2.5 (0.42)	2.3 (0.32)			
PHQ‐9 (0–27)			0.12	1.97	0.87
V0	2.5 (0.41)	2.9 (0.36)			
V2	2.6 (0.39)	2.8 (0.41)			
V3	2.8 (0.52)	2.4 (0.31)			
V4	2.0 (0.31)	2.4 (0.36)			
V5	2.2 (0.37)	2.2 (0.38)			
HADS‐A (0–21)			2.52	13.67****	1.98
V0	5.0 (0.56)	6.3 (0.44)			
V2	4.0 (0.51)	4.2 (0.43)			
V3	3.5 (0.43)	4.0 (0.34)			
V4	3.0 (0.37)	4.3 (0.43)			
V5	3.3 (0.45)	4.2 (0.43)			
FCRI‐Severity (0–36)			0.63	13,90****	1.58
V0	10.5 (0.4)	12.9 (0.83)			
V2	10.0 (0.77)	10.9 (0.74)			
V3	8.9 (0.74)	8.8 (0.73)			
V4	8.2 (0.73)	8.4 (0.71)			
V5	8.4 (0.73)	8.6 (0.73)			
ISI (0–28)			0.76	4.86***	0.35
V0	6.7 (0.67)	7.3 (0.67)			
V2	6.2 (0.72)	7.5 (0.68)			
V3	5.8 (0.64)	6.4 (0.63)			
V4	5.3 (0.61)	5.9 (0.60)			
V5	6.0 (0.69)	6.3 (0.68)			
FSI‐Severity (0–30)			0.34	1.51	0.51
V0	7.9 (0.68)	8.3 (0.68)			
V2	7.6 (0.65)	8.0 (0.61)			
V3	7.4 (0.62)	7.3 (0.60)			
V4	6.6 (0.60)	7.6 (0.58)			
V5	7.6 (0.70)	8.0 (0.68)			
FSI‐Impact (0–70)			0.00	1.34	2.43
V0	9.1 (1.35)	8.9 (1.13)			
V2	10.0 (1.45)	9.3 (1.14)			
V3	9.6 (1.44)	7.6 (0.88)			
V4	7.0 (0.91)	8.8 (1.28)			
V5	8.0 (1.34)	9.6 (1.42)			
FSI‐No of days (0–7)			1.33	2.48*	0.84
V0	2.0 (0.26)	2.5 (0.25)			
V2	1.9 (0.28)	2.5 (0.28)			
V3	1.9 (0.25)	2.0 (0.25)			
V4	1.7 (0.26)	2.0 (0.26)			
V5	2.1 (0.28)	2.3 (0.28)			
FSI‐Extent (0–10)			1.05	0.81	1.18
V0	2.0 (0.26)	2.2 (0.26)			
V2	1.7 (0.23)	2.0 (0.21)			
V3	1.8 (0.20)	1.8 (0.20)			
V4	1.7 (0.22)	2.2 (0.21)			
V5	1.8 (0.22)	2.1 (0.21)			
FACT‐Cog‐PCI (0–72)			0.13	0.65	1.53
V0	61.9 (1.2)	61.8 (1.2)			
V2	62.8 (1.3)	61.4 (1.3)			
V3	61.1 (1.3)	62.0 (1.3)			
V4	62.5 (1.3)	61.3 (1.3)			
V5	63.0 (1.3)	61.9 (1.3)			
FACT‐Cog‐Others (0–16)			0.06	0.52	1.24
V0	15.6 (0.20)	15.4 (0.18)			
V2	15.5 (0.15)	15.5 (0.18)			
V3	15.5 (0.16)	15.7 (0.11)			
V4	15.7 (0.11)	15.5 (0.20)			
V5	15.7 (0.12)	15.6 (0.15)			
FACT‐Cog‐PCA (0–28)			0.01	0.61	0.15
V0	22.7 (0.86)	22.5 (0.85)			
V2	21.7 (0.95)	22.2 (1.03)			
V3	22.6 (0.91)	22.7 (0.94)			
V4	23.1 (0.81)	22.5 (0.92)			
V5	23.2 (0.82)	22.9 (0.91)			
FACT‐Cog‐Impact (0–16)			0.03	3.03*	3.39*
V0	14.2 (0.40)	14.0 (0.40)			
V2	13.5 (0.45)	13.9 (0.42)			
V3	14.4 (0.28)	15.0 (0.27)			
V4	14.8 (0.28)	14.4 (0.28)			
V5	14.6 (0.36)	14.5 (0.36)			

Abbreviations: Extent, how much of the day they felt fatigued; FACT‐Cog, Functional Assessment of Cancer Therapy—Cognitive Function; FCRI, Fear of Cancer Recurrence Inventory; FSI, Fatigue Symptom Inventory; HADS‐A, anxiety subscale of the Hospital Anxiety and Depression Scale; HADS‐D, depression subscale of the Hospital Anxiety and Depression Scale; Impact, impact on quality of life; ISI, Insomnia Severity Index; Others, comments from others; PCA, perceived cognitive abilities; PCI, perceived cognitive impairment; PHQ‐9, Patient Health Questionnaire‐9; V0, baseline; V2, 3 months; V3, 6 months; V4, 9 months; V5, 12 months.

*Note*: *p < 0.05; **p < 0.01; ***p < 0.001; ****p < 0.0001.

The FACT‐Cog‐Impact subscale was the only variable on which a significant group‐by‐time interaction was obtained, but post hoc analyses indicated a significant change in the placebo group only with a greater score at V3 (*M* = 15.02) as compared to V0 (*M* = 13.99), V2 (*M* = 13.93), and V4 (*M* = 14.36), *F* (4, 123.4) = 5.07, *p* < 0.001. Of note, effect sizes (ES) were calculated on the group‐by‐time interaction effects obtained comparing V0 and V5, on each study variable. Except for *Fear of Cancer Recurrence Inventory* (FCRI) scores (ES = 0.36), effect sizes obtained were all smaller than 0.20, thus all falling in the small effect range. Moreover, for FCRI, scores reduction between V0 and V5 indicated a larger effect in the placebo than in the intervention group.

### 3.3 Treatment effects on proportion of patients with clinical levels of symptoms

Generalized linear mixed models revealed significant time effects on the proportion of patients having a clinical anxiety (HADS‐A) and FCR (FCRI) score. Post hoc comparisons showed that the proportion of patients with clinical anxiety was significantly greater at V0 (30.6%) as compared to V3 (12.6%) and V5 (16.1%) and greater at V2 (25.4%) than V3 (12.6%). In addition, they revealed proportions of clinically significant FCR which were significantly higher at V0 (42.2%) than V3 (26.3%), V4 (25.9%), and V5 (23.6%). No significant time effect was found on proportions of patients with clinical depression (HADS‐D, PHQ‐9), insomnia (ISI), fatigue (FSI), cognitive impairments (FACT‐Cog), and no significant group‐by‐time interaction was obtained on any variable.

### 3.4 Treatments effects on psychological variables (mean scores) by presence/absence of clinical levels of symptoms at baseline (see Data S1 for details)

No significant group, time, or interaction effect was observed on PHQ‐9 and FACT‐Cog scores as a function of the presence/absence of clinical levels at baseline. HADS‐D scores significantly improved over time in patients with clinically meaningful depression at baseline, but not in patients without a clinical level of depression. However, there was no difference in whether patients had received MAG‐EPA or placebo. For HADS‐A, FCRI, ISI, and FSI, a significant change over time was found both in those with a clinical level and those with a nonclinical level at baseline. Again, there was no difference between MAG‐EPA and placebo groups.

### 3.5 Sensitivity analyses

Four (7.1%) of the MAG‐EPA patients received adjuvant treatment (i.e., hormone therapy and/or radiation therapy) during the course of the study, as compared to three patients (5.0%) in the placebo group. In addition, 7 MAG‐EPA patients (12.5%) experienced disease progression (e.g., PSA ≥ 0.2 ng/mL post‐RP, biochemical relapse), as compared to four patients (6.7%) in the placebo group. Sensitivity analyses excluding these patients did not significantly alter the study findings, nor its conclusions.

## 4 DISCUSSION

The goal of this planned secondary analysis of a one‐year phase IIb RCT was to evaluate the effects of MAG‐EPA supplementation as compared to those of a placebo (HOSO) on depression and other psychological symptoms that are prevalent in men with prostate cancer. Findings revealed significant changes over time of several variables, that is depression (assessed with HADS‐D but not PHQ‐9), anxiety, FCR, insomnia, fatigue (number of days), and cognitive functioning (impact), mainly at the beginning of the study. However, patients who received MAG‐EPA did not show significantly greater changes than those on placebo on any variable and at any time point. Omega‐3 supplementation does not appear to improve depression and other patient‐reported outcomes in men with prostate cancer treated with RP.

Comparing results with the previous literature is challenging given significant differences in populations studied, and the dosage and composition of the n‐3 PUFA used (e.g., proportion and dosage of EPA and DHA). Yet, results of this trial contradict prior evidence showing reduced depression with n‐3 supplementation in the general population despite the fact that our supplements contained a larger amount of EPA and a greater EPA:DHA ratio than most prior studies.^13^, ^14^, ^15^ To our knowledge, no other clinical trial has been conducted on the effect of n‐3 supplementation on depression in the context of cancer. However, our results are consistent with those of Kiecolt‐Glaser et al.'s RCTs conducted in medical students^45^ and middle‐aged and older adults^17^ also having low levels of depression at baseline and using a similar dosage (2.5 g/day; 2085 mg EPA and 348 mg DHA). Together, this could suggest that our participants were not symptomatic enough for us to be able to detect a therapeutic effect (i.e., floor effect). However, no greater effect of MAG‐EPA over placebo was found either in patients with clinical levels of depression at baseline when analyzed distinctly from those without. In fact, despite having low levels of depression on average at baseline, participants showed a general decrease in depressive symptoms whether they received n‐3 supplementation or not and whether they had or not clinically significant depressive symptoms at baseline, although to a larger extent in those with a clinical level. Interestingly, the most recent Cochrane review of clinical trials on the effects of n‐3 PUFAs on depression, which reviewed studies conducted in various populations, including some with adults having a medical comorbidity (e.g., cardiovascular disease), suggested that there may be a “small‐to‐modest benefit” for depressive symptoms that is “unlikely to be clinically meaningful for patients with a major depressive disorder.”^46^ Overall, it may be concluded that improvements in depression symptoms observed in our study were most likely due to non‐specific therapeutic factors such as regression to the mean or passage of time.

Prior evidence on effects of n‐3 supplementation on other symptoms was rather sparse and equivocal. Consistent with RCTs conducted in individuals without cancer,^16^, ^17^, ^47^ the current study did not find a greater effect of MAG‐EPA over placebo on insomnia in men with prostate cancer. However, our results contrast with promising results obtained for the treatment of anxiety disorders^18^ and cognitive functioning^19^ in the general population and cancer‐related fatigue.^25^, ^26^, ^27^ Indeed, omega‐3 supplementation was not associated with significantly greater improvements in anxiety and fatigue as compared to placebo in this study, and for cognition, the only significant group‐by‐time interaction was on FACT‐Cog‐Impact scores. This result could be due to multiple testing and be attributable to chance only, but more importantly, simple effects indicated that a significant improvement in FACT‐Cog‐Impact was found only in the placebo group. As these symptoms were highly prevalent at baseline, the lack of between‐group differences in changes over time cannot be attributed to a floor effect. Another possible explanation for the lack of larger effects for MAG‐EPA, especially on cognitive functioning, could be related to the content of capsules used, which were composed of 80% of EPA and less than 10% of DHA. DHA is the main fatty acid in the brain, and supplements of DHA at a dosage around 1800 mg/day have been found to be related to improved cognitive function in the general population.^23^, ^24^ Also, we cannot exclude a possible beneficial effect of the placebo, composed of vegetal oil rich in n‐9.^48^ However, HOSO is virtually neutral on inflammation (no inflammatory metabolites have been identified) and has been used as a placebo in many previous clinical trials.^49^, ^50^, ^51^ Again, it would appear that the effect of nonspecific factors (e.g., passage of time) constitutes the most plausible explanation for improvements that occurred over time in anxiety, FCR, insomnia, and fatigue in the current study.

It is noteworthy that RP took place between V0 and V2 when most of the significant reductions were found. Longitudinal studies have shown that depression, FCR, and symptoms such as insomnia and perceived cognitive impairments are at their highest point around the time of cancer diagnosis and improve afterwards.^6^, ^52^, ^53^, ^54^, ^55^ Although such natural remission may appear reassuring, it remains important to offer effective interventions to patients with significant psychological symptoms. Indeed, a significant proportion of patients still had clinical levels of anxiety (MAG‐EPA: 14.2%; placebo: 18.3%), FCR (MAG‐EPA: 23.5%; control: 23.7%), insomnia (MAG‐EPA: 31.5%; placebo: 31.7%), and fatigue (MAG‐EPA: 37.6%; placebo: 40.9%) 12 months following RP. All of these symptoms and even more when they are comorbid, are likely to negatively impact patients' quality of life and daily functioning.^56^, ^57^, ^58^

This study has several strengths. Patients were recruited in routine cancer care thus increasing the study's external validity. Symptoms were measured using validated self‐report scales. The one‐year follow‐up with multiple time points and the use of placebo capsules identical in appearance to MAG‐EPA capsules are other strengths. On the other hand, our participants had a circulating n‐6/n‐3 ratio in red blood cell membranes of 3.7 ± 0.68 (Western diet is typically around 16:1^59^) at baseline, thus possibly limiting the benefits that they could get from n‐3 supplementation. Also, the study was initially powered to detect significant between‐group differences on a biological outcome and effect sizes of at least a moderate magnitude.^29^ Effect sizes of group‐by‐time interactions obtained on psychological outcomes were all of a much smaller magnitude (V0 vs V5: smaller than 0.20) and, when they were larger, they were not in the expected direction (favored the placebo group), thus limiting the likelihood that the lack of superiority of MAG‐EPA was due to the small sample size. The lack of selection of participants based on significant symptoms levels is another weakness, which is however due to the secondary nature of this analysis. In particular, the small proportion of patients having clinically significant depression at study entry reduced the statistical power and capacity to test decisively the antidepressant effect of MAG‐EPA in this population. In addition, the randomization failed to allocate participants equally to the two study groups on some relevant variables (i.e., NCCN risk classification, biopsy Gleason score, baseline EPA levels). Although a statistical control was performed, this remains a limitation as it raises questions as to whether an imbalance might have occurred on other known and unknown confounding variables, which would threaten the validity of the results. Lastly, although counts of pills not used and returned to the pharmacy did not differ across groups (compliance of 93.3% and 91.9% in MAG‐EPA and placebo groups, respectively),^30^ the extent to which participants actually took the prescribed capsules is uncertain. However, the measure of EPA levels in red blood cell membranes suggests an adequate compliance.^30^, ^31^

In conclusion, results of this trial did not support the efficacy of n‐3 supplementation in alleviating depression, anxiety, FCR, insomnia, fatigue, and subjectively assessed cognitive impairments in men treated for prostate cancer with RP. RCTs that specifically include patients with clinical levels of depression and other symptoms are warranted.

## AUTHOR CONTRIBUTIONS

**Josée Savard:** Conceptualization (supporting); data curation (equal); formal analysis (lead); funding acquisition (equal); methodology (equal); validation (equal); visualization (equal); writing – original draft (lead); writing – review and editing (lead). **Hanane Moussa:** Data curation (supporting); writing – original draft (supporting); writing – review and editing (supporting). **Jean‐François Pelletier:** Data curation (supporting); writing – original draft (supporting); writing – review and editing (supporting). **Pierre Julien:** Data curation (supporting); investigation (supporting); methodology (supporting); writing – original draft (supporting); writing – review and editing (supporting). **Louis Lacombe:** Data curation (supporting); investigation (supporting); methodology (supporting); writing – original draft (supporting); writing – review and editing (supporting). **Rabi Tiguert:** Investigation (supporting); methodology (supporting); writing – original draft (supporting); writing – review and editing (supporting). **Yves Caumartin:** Data curation (supporting); investigation (supporting); writing – original draft (supporting); writing – review and editing (supporting). **Thierry Dujardin:** Data curation (supporting); investigation (supporting); writing – original draft (supporting); writing – review and editing (supporting). **Paul Toren:** Data curation (supporting); investigation (supporting); writing – original draft (supporting); writing – review and editing (supporting). **Pouliot Frédéric:** Data curation (supporting); investigation (supporting); writing – original draft (supporting); writing – review and editing (supporting). **Michele Lodde:** Data curation (supporting); investigation (supporting); writing – original draft (supporting); writing – review and editing (supporting). **Yves Fradet:** Data curation (supporting); investigation (supporting); writing – original draft (supporting); writing – review and editing (supporting). **Karine Robitaille:** Conceptualization (supporting); data curation (supporting); formal analysis (supporting); funding acquisition (supporting); investigation (supporting); methodology (supporting); project administration (supporting); resources (equal); supervision (supporting); writing – original draft (supporting); writing – review and editing (supporting). **Vincent Fradet:** Conceptualization (equal); data curation (equal); formal analysis (supporting); funding acquisition (lead); investigation (lead); methodology (lead); project administration (lead); resources (lead); supervision (lead); writing – original draft (supporting); writing – review and editing (supporting).

## FUNDING INFORMATION

This work was mainly supported by the Canadian Cancer Society (Grant #702569). It was also supported by the Foundation of CHU de Québec‐Université Laval. VF and HM are supported by a Fonds de recherche du Québec—Santé (FRQS) clinician scientist career grant and doctorate scholarship, respectively.

## CONFLICT OF INTEREST STATEMENT

The authors declare no conflict of interest related to this work.

## CLINICAL TRIAL REGISTRATION

NCT02333435.

## Supporting information


Data S1.

## Data Availability

The dataset used and analyzed during the current study is available from the corresponding author on reasonable request

## Footnotes

* The term « psychological symptoms » is utilized inclusively here to designate symptoms that are often considered somatic in nature but have a strong psychological component.
