# Supplementary material for: Effects of omega‐3 supplementation on psychological symptoms in men with prostate cancer: Secondary analysis of a double‐blind placebo‐controlled randomized trial
Source: Cancer Med. 2023 Oct 3;12(19):20163–76. doi: 10.1002/cam4.6598 (PMC10587967; doi:10.1002/cam4.6598)
Supplement: Supplementary file 1 — Data S1. [file CAM4-12-20163-s001.docx]

Supporting Information

*More Specific Information on Treatment Effects on Psychological Variables (Mean Scores) by Presence/Absence of Clinical Levels of Symptoms at Baseline*

*Depression.* No significant group, time or interaction effect was found on PHQ-9 as a function of the presence/absence of clinical levels at baseline. HADS-D scores significantly improved over time in patients with clinically meaningful depression at baseline, *F*(4, 9.98)=15.74, *p*<.001, but not in patients without a clinical level of depression, *F*(4, 167.1)=1.54, *p*=0.19. Scores of patients with a clinical level of depression were significantly greater at V0 (M=9.53) as compared to V2, V3 and V4 (M=5.16, 4.30, 4.01, respectively). However, there was no difference on the evolution of depression scores whether patients had received MAG-EPA or placebo.

*Other variables.* For several variables, there was a significant change over time both in patients with clinically significant symptoms at baseline [HADS-A: *F*(4, 22.73)=55.18, *p*<.0001; FCRI: *F*(4, 46.13)=17.98, *p*<.0001; ISI: *F*(4,44.84)=7.07, *p*<.001; FSI: *F*(4,78.09)=12.98, *p*<.0001] and in those with a non-clinical level [HADS-A: *F*(4,83.86)=3.13, *p*<.05; FCRI: *F*(4, 54.7)=4.06, *p*<.01; ISI: *F*(4, 76.19)=3.36, *p*<.05; FSI: *F*(4, 90.11)=5.90, *p*<.0001]. Again, there was no difference between MAG-EPA and placebo groups on the evolution of symptoms.

More specifically, in patients with a clinical level of anxiety at baseline, anxiety scores were significantly higher at V0 (M=10.70) than at the subsequent time points (M=6.88, 6.16, 5.68, 6.53, respectively), while in those with a non-clinical level, scores were significantly higher at V0 (M=3.51) than at V3 (M=2.81) and V5 (M=2.69). FCR scores significantly decreased between V0 (M=17.72) and V2 (M=14.31) and between V2 and all subsequent time points (M=11.67, 11.44, 11.96, respectively) in those with a clinical FCR level at baseline and between V2 (M=7.74) and V4 (M=6.04) in those without. A significant reduction of insomnia scores was found between V0 (M=12.56) and all subsequent time points (M=9.78, 9.44, 9.00, and 9.45, respectively) in patients with clinical insomnia at baseline, while a significant difference was found between V2 (M=5.07) and V0 (M=3.46) and V4 (M=3.38) in those without. Patients with a clinical level of fatigue at V0 showed a significant decline in FSI scores between baseline (M=4.29) and all subsequent time points (M=3.09, 3.23, 3.03, and 3.23, respectively) while those without clinically significant fatigue showed a significantly higher fatigue at V2 (M=2.17) and V4 (M=1.77) as compared to V0 (M=1.36).

No significant group, time or interaction effect was found on FACT-Cog scores as a function of the presence/absence of clinical levels at baseline.
